# The PhysioCam: A Novel Non-Contact Sensor to Measure Heart Rate Variability in Clinical and Field Applications

**DOI:** 10.3389/fpubh.2017.00300

**Published:** 2017-11-22

**Authors:** Maria I. Davila, Gregory F. Lewis, Stephen W. Porges

**Affiliations:** ^1^Brain Body Center for Psychophysiology and Bioengineering (BBCPB), Department of Psychiatry, University of North Carolina at Chapel Hill, Chapel Hill, NC, United States; ^2^Intelligent Systems Engineering, Indiana University Bloomington, Bloomington, IN, United States; ^3^Kinsey Institute, Indiana University Bloomington, Morrison Hall Indiana University, Bloomington, IN, United States

**Keywords:** heart rate variability, non-contact monitoring, arterial pulse, sensors agreement, optics and physiology

## Abstract

Heart rate variability (HRV) is a reliable indicator of health status and a sensitive index of autonomic stress reactivity. Stress negatively affects physical and psychological wellness by decreasing cardiovascular health and reducing quality of life. Wearable sensors have made it possible to track HRV during daily activity, and recent advances in mobile technology have reduced the cost and difficulty of applying this powerful technique. Although advances have made sensors smaller and lighter, some burden on the subject remains. Chest-worn electrocardiogram (ECG) sensors provide the optimal source signal for HRV analysis, but they require obtrusive electrode or conductive material adherence. A less invasive surrogate of HRV can be derived from the arterial pulse obtained using the photoplethysmogram (PPG), but sensor placement requirements limit the application of PPG in field research. Factors including gender, age, height, and weight also affect PPG-HRV level, but PPG-HRV is sufficient to track individual HRV reactions to physical and mental challenges. To overcome the limitations of contact sensors, we developed the PhysioCam (PhyC), a non-contact system capable of measuring arterial pulse with sufficient precision to derive HRV during different challenges. This passive sensor uses an off the shelf digital color video camera to extract arterial pulse from the light reflected from an individual’s face. In this article, we validate this novel non-contact measure against criterion signals (ECG and PPG) in a controlled laboratory setting. Data from 12 subjects are presented under the following physiological conditions: rest, single deep breath and hold, and rapid breathing. The following HRV parameters were validated: interbeat interval (IBI), respiratory sinus arrhythmia (RSA), and low frequency HRV (LF). When testing the PhyC against ECG or PPG: the Bland–Altman plots for the IBIs show no systematic bias; correlation coefficients (all *p* values < 0.05) comparing ECG to PhyC for IBI and LF approach 1, while RSA correlations average 0.82 across conditions. We discuss future refinements of the HRV metrics derived from the PhyC that will enable this technology to unobtrusively track indicators of health and wellness.

## Introduction

Heart rate variability (HRV) frequently has been applied as a reliable indicator of health status, stress, and mental effort. Studies have linked HRV to cardiovascular diseases (1), post-traumatic stress disorder (2), depression (3), and fibromyalgia (4). HRV has been proposed as a sensitive index of autonomic stress reactivity such as in panic disorder (5) and work stress and mental effort (6). The literature on HRV indicates that day-to-day challenges that increase stress and reduce HRV have a negative influence on physical and psychological wellness by decreasing cardiovascular health and reducing quality of life.

Advances in technology have created the opportunity to apply wearable sensors to track HRV during daily activity (7, 8). Movement and light sensors embedded on mobile phones are used as contact sensors to acquire HRV indices (9, 10). These improvements in sensors, coupled with recent advances in mobile technology, have reduced the cost and difficulty of monitoring HRV outside the laboratory in applied contexts. Although sensors are smaller and lighter, they still impose a burden on the human subject. Chest-worn electrocardiogram (ECG) sensors provide the optimal source signal for HRV analysis, but they require an obtrusive electrode or conductive material adherence. A less invasive surrogate of HRV can be derived from the arterial pulse obtained using the photoplethysmogram (PPG), which provides sufficient accuracy to track individual HRV reactions to physical and mental challenges (11). However, sensor placement requirements limit the application of PPG in field research and participant awareness of being monitored is required with both contact sensors (i.e., ECG and PPG).

This article describes an innovative technology embodied in a novel device, the PhysioCam (PhyC) (12) that overcomes the limitations of contact sensors. The PhyC is a non-contact system capable of measuring arterial pulse with sufficient precision to derive HRV during different physiological challenges. In this article, we explore the science behind the PhyC, the implications of measuring HRV from the vascular periphery, and the future of non-contact sensors.

Non-contact technology is an emerging technology; recent research has explored the use of a basic webcam for measuring multiple physiological parameters. Poh et al. (13) used an inexpensive webcam with automatic face tracking and blind source separation of the color channels into independent components to extract cardiac pulse rate from a video recording. They applied a fast Fourier transform analysis to a 1-min recording of the video to extract the predominant frequency of variation corresponding to the cardiac pulse rate. Subsequently, the same team (14) reported the possibility to obtain the low frequency HRV and respiratory sinus arrhythmia (RSA) components of HRV applying blind source separation and frequency analysis of the components. Phillips released the Vital Signs Camera for iPad 2 or iPhone 4S, an application that uses the webcam capabilities of the tablets to acquire parameters of heart rate and breathing rate from a user who is sitting still in front of the camera. More than 80 applications have been developed that enable smartphone cameras to monitor heart rate. However, these applications use the phones light source and require physical contact with the fingertip. The commercially available applications do not disclose their approach to measure heart rate or breathing rate, but they employ some form of frequency analysis of digital camera data.

Research into non-contact extraction of physiological signals extends beyond pulse measurements with infrared video thermography being employed to accurately estimate breathing rate and relative tidal volume (15) and various forms of radar being explored to locate respiratory signals as well as cardiac activity (16, 17). Wu et al. (18) presented a method to visualize the flow of blood using a standard video sequence as input and applying spatial decomposition, followed by temporal filtering of the frames. The analysis reveals temporal variations in videos that cannot be seen by the naked eye and could represent blood flow; they called their method Eulerian Video Magnification. An evaluation of the literature suggests that non-contact extraction of human physiological parameters is both feasible and of considerable interest for research and commercial applications.

Current approaches to non-contact measures of HRV have limitations, since it is essential to have a high-quality pulse signal and a sensitive algorithm to detect features in the pulse signal to accurately time the sequential interbeat intervals (IBIs) and provide a valid and accurate estimate of IBIs derived from the criterion ECG signal.

In the case of non-contact sensors that utilize videography, the light sensor embedded in the camera limits the quality of the pulse signal. Most video cameras use one of two types of light sensors, the charge-coupled device (CCD) and the complementary metal-oxide semiconductor (CMOS). These photosensors transform light into electrons by the same approach, but use different methods to digitize the information. The CCD sensor digitization process produces high-quality and low-noise images in contrast to the CMOS sensor, which has greater interdependence of pixel level information from surrounding portions of the image array (19), making the CCD sensor an appropriate choice when analyzing biological signals through a Bayer-mapped pixel array.

To optimize frame-by-frame extraction of pulse information to facilitate IBI measurements, we selected a CCD sensor for the PhyC. Conceptually, the frame-by-frame extraction functions similar to digitizing a PPG signal. By contrast, the work of Poh and others is based on webcam with CMOS sensors. As noted, CMOS sensors have greater crosstalk between adjacent pixels of different color sensitivities (e.g., red or green). This may explain why blind source separation is required to extract the underlying physiological variance in the capture image sequences (13, 14).

The PhyC uses a CCD camera as its sensor and can function in ambient (sun, fluorescent, or incandescent) light. The CCD camera operates continuously, capturing images of the subject from a distance of about 3 m, with a field of view that encompasses the shoulders, neck, and face of the individual. From the collected images, a subset of pixels is extracted in each frame that contains the skin area of the upper half of the face. The 3-m distance from PhyC to the target subject in this study is determined by optics, number of pixels of the sensor, ambient light, and area of the body from which the signal is being detected. This distance could be modified for different applications.

The optical properties of human skin are determined by the presence of various chromophores in the layers of skin. The epidermis and the dermis are the predominant layers responsible for the optical properties of the skin. The epidermis is the outermost layer of skin, with a thickness varying from 0.05 to 1.50 mm. The main chromophore in the epidermis is melanin and it is essentially the only pigment affecting the transmittance of light in normal human epidermis (20). The dermis is the intermediate layer of the skin with a thickness varying from 0.3 to 3.0 mm. The predominant chromophores in the dermis are the blood-borne pigments hemoglobin, oxyhemoglobin, beta carotene, and bilirubin. Each of the pigments has different absorption spectra in the range of visible light (from 300 to 1,200 nm). The innermost and thickest layer of the skin is the hypodermis, which is connected to the dermis by collagen and elastin fibers. In between the dermis and the hypodermis is the arteriovenous plexus. The superficial arteriovenus plexus forms a microvascular network typically 0.05–0.50 mm below the skin surface (21) and the deep arteriovenus plexus forms a vascular network typically 1.00–5.00 mm below the skin surface. Thus, the main chromophores determining skin color are the melanin present in the epidermis and the blood-borne pigments present in the dermis/hypodermis vascular plexus.

**Figure 1:**
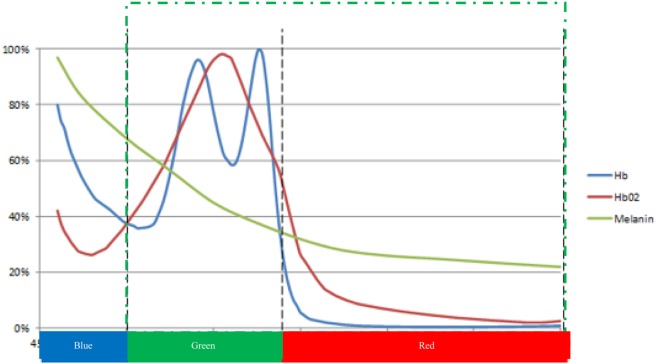
Schematic representation of the relative light absorption of the main chromophores of the human skin: Hb (deoxygenated hemoglobin), HbO_2_ (oxygenated hemoglobin), and melanin. Bottom color bar: spectral response range for digital video cameras [adapted from Prahl (23) and Huang et al. (24)].

Within the pixels selected to be processed, the optical characteristics of hemoglobin, an active chromophore in the visible light range, determine the wavelengths of interest. Given the high percentage of hemoglobin in the blood composition (around 45%) (22), it is possible to measure the volumetric blood changes by a light sensor that works in the visible light range (see Figure 1). The PhyC decomposes consecutive images to extract the subtle volumetric blood changes from light reflectance changes (i.e., observed color). In addition to the physiological (blood volume) sources of variation in light intensity within these pixels, there are a number of sources of noise in the signal, including inconsistent illumination intensity, movement, shadow, skin color, electrical noise, and failure of the motion tracking algorithms to reliably identify the relevant pixels.

The camera measures the segment of light in the visible band, the portion of the electromagnetic spectrum in the region from approximately 380 to 775 nm. The light captured by the camera is segmented using the red, green, and blue (RGB) Bayer filter pattern into three sub bands: blue (380–500 nm), green (500–600 nm), and red (600–775 nm). The optical properties of the skin, blood, and CCD sensor enable the PhyC to function as a biosensor of cardiovascular activity at the surface of the face.

Figure 1 shows the absorption curve of the hemoglobin (red and blue lines) in relative units. The normalized, relative melanin absorption curve (green line) is superimposed on the hemoglobin absorption curve. At the bottom of the *X*-axis is the range of the spectral response curves for the three color bands of the CCD sensor. Melanin accounts for skin color and predominantly acts in the short wavelength range of the visible light (<500 nm). In this range, differences in skin colors affect the light absorption curve. In the longer wavelength range of light (i.e., above 500 nm), melanin has a notably smaller, more consistent impact on absorbance. The PhyC utilizes wavelengths longer than 500 nm to minimize between subject differences in signal strength due to skin color, green dotted section on Figure 1. Only green (500–575 nm) and red (575–750 nm) pixel values are used to estimate relative absorption of light by the blood.

The PhyC utilizes an algorithm integrating knowledge of the physiology of the arterial pulse wave with the capabilities of the camera. The natural or artificial light illuminates the person’s face, and some of the light is transmitted through the epidermis and penetrates the skin about 2–3 mm deep into the dermis, where the different components of the dermis reflect, absorb, and/or transmit the light. The main source of rhythmic variation in light absorbance and reflectance in the dermis is the hemoglobin present in the blood vessels. The volume of blood in the arteries and arterioles changes as a function of the beating of the heart. Each heart beat generates a pressure wave that changes the radius of arteries and arterioles. Volumetric changes in the arterial bed cause reflectance and absorbance changes along the transmission and reflection pathways of the incident light. The video camera captures the light reflected by the person’s face and the subtle changes in that reflected light due to pulse wave activity. When there is more blood in the arteries and arterioles and more light is absorbed by the blood, the camera detects less reflected red and green light. On the contrary, when there is less blood in the arteries and arterioles, the blood absorbs less light and the camera detects more reflected light (25).

This report evaluates the validity of the derived video pulse wave by comparing it with criterion signals. The precision of PhyC derived beat-to-beat intervals (IBI) were evaluated by contrasting these values with contact measures from an ECG and earlobe photoplythesmogram (elPPG). In addition, derived variables of average heart period (HP), RSA, and low frequency HRV (LF) during specific experimental conditions were contrasted.

## Materials and Methods

### General Approach

Heart rate variability is defined as the variations in time between sequential heartbeats. When HRV is deconstructed through statistical procedures, it is possible to quantify rhythmic components that reflect specific pathways of neural regulation. The most salient components are a respiratory oscillation known as RSA [e.g., Ref. (26)] and a slower frequency (LF) assumed to be related to blood pressure regulation *via* the baroreceptors and peripheral vasomotor activity [for overview see Porges (27) and Reyes del Paso et al. (28)].

Respiratory sinus arrhythmia is assumed to reflect cardiac vagal tone *via* myelinated pathways originating in a brainstem area known as nucleus ambiguus. RSA is due specifically to myelinated vagal efferent fibers and the other HRV components are not specific, and thus may not include both myelinated and unmyelinated vagal fibers. The unmyelinated vagal efferent fibers originate in a brainstem area known as the dorsal nucleus of the vagus and may contribute to the lower frequencies of HRV. Removing RSA from HRV might result in a metric that would be mediated by a contribution of both dorsal and ventral vagal influences. Blockade studies are not useful in differentiating the influence of myelinated and unmyelinated vagal pathways on HRV, since virtually all HRV (i.e., RSA and lower frequencies) is removed with muscarinic blockade (e.g., atropine) (29–31). In addition, HP (i.e., average IBI over a period of time) was monitored, since it reflects the sum of neural, neurochemical, and intrinsic influences on the heart.

The literature, identifying neurophysiological mechanisms and sensitivity of HRV components to behavioral, psychological, and health parameters, is primarily based on the analysis of the heart rate patterns measured with an ECG sensor. ECG has been the signal of choice, since the sequence of times between R-peaks can provide a non-invasive (but not non-contact) measure of the neural regulation of the heart (32).

The PhyC and the elPPG measure heart rate at the periphery, where measures of beat-to-beat heart rate contain the source information from the ECG with added sources of variance. The main source of variance is the vascular system, which acts as a filter modulating the propagation of the heart beat and introducing an additional source of variations in the timing between the sequential pulse-to-pulse interval time series (33). The inherent functioning of the elPPG and the PhyC as photo sensors further introduces movement as a potential source of measurement error. The non-contact nature of the PhyC amplifies the measurement error due to greater variations in movement and light source and a slower sampling frequency of the sensor. To validate the variables derived by the PhyC, an experiment was conducted to identify, describe, and understand the similarities and differences among the values obtained by PhyC, elPPG, and ECG.

### Experiment Design

To test the PhyC, we designed an experiment consisting of a resting baseline period followed by several challenges designed to alter the neural regulation of the heart that would be manifested in changes in HRV. By presenting different challenges, it was possible to evaluate whether the PhyC accurately tracked the physiological changes monitored *via* ECG and elPPG.

### Physiological Challenges

- Breathing rate: voluntary or involuntary respiratory rate shifts the neural influence of vagal pathways on heart rate and RSA (34). To evaluate the capability of the PhyC to track changes in autonomic state caused by shifts in respiration rate, two breathing patterns were used (single deep breath and sustained rapid shallow breathing). Single deep breath and hold (SDB) was done by inhaling and holding the volume of air for as long as possible follow by normal breathing. The SDB is accompanied by decreases in heart rate and RSA (34). Shallow rapid breathing (RB) was implemented by restricting the volume of air inhaled on each breath. Shallow breathing decreases the amplitude of the RSA and increases heart rate (35).
- Cold pressor (CP): the CP test consisted of the immersion of a hand and forearm in ice water for 90 s or as long as tolerated by the participant (36). Due to signal distortions from movement and variations in the duration, immersion data from this condition were not examined for this paper.

### Participants

This study was approved by the Institutional Review Board of the University of Illinois at Chicago as protocol # 2012-0206 entitled “Real Time Non-contact Extraction of Human Arterial Pulse.” The IRB authorized the recruitment of 20 subjects through flyers, the University of Illinois at Chicago Psychology student subject pool, and *via* email to the UIC students’ community. Twenty participants were recruited (10 females and 10 males) between 19 and 71 years of age with a mean of 33.13 years and SD of 14.62 years. 17 subjects reported no preexisting medical condition. Three females had specific medical conditions: one was 6 months pregnant, one was diagnosed with multiple sclerosis and syncope, and one reported an undiagnosed vascular constriction condition. The ethnicity mix of our participants was 60% Caucasians, 25% Hispanics, 10% African-Americans, and 5% Asians.

Data from three subjects were not analyzed for the following reasons: one criterion signal was corrupted making it impossible to access both the ECG and elPPG files, one participant presented with an extreme tonic peripheral vasoconstriction that masked the pulse wave precluding the ability to extract the arterial pulse by the PhyC, and one participant presented sufficiently low HRV that precluded the calculation of the LF component. Data from the remaining seventeen subjects were processed for analysis. Movement-related confounds in some of the criterion variables resulted in an exclusion of segments of data for some participants during specific challenges. For this article, the 12 participants with complete sets of data for the 5 reported tasks are included.

The protocol as approved by the IRB contained two stages:

- Offline: during the offline stage, video images were recorded and then processed to develop and to optimize the algorithms to process the video signal. The offline stage lasted 15 min, during which participants were asked to perform the following experimental manipulations:
  - Initial rest baseline (2 min) (1BSL).
  - SDB (2 min).
  - Rest (2 min) (2BSL).
  - CP.
  - CP recovery (approximately 3 min) (CPr).
  - Rest (1 min) (3BSL).
  - Shallow RB (2 min).
  - Rest (3 min) (4BSL).
- Online (proof of concept): during the online stage, the PhyC extracted the arterial pulse wave in near real time. The objective of the online stage was to evaluate the factors that may limit applications of the technology such as head and face tracking, processing demands, and time delay between acquisition and output. During this stage, participants were asked to sit quietly during 5 min while the arterial pulse wave was monitored. Data from this stage are not reported in this paper, because of two limiting factors: (1) the slow sampling frequency (30 Hz) at which the signal was collected resulted in greater variations in the estimates of IBIs from the pulse wave and (2) in several participants, the system could not track continuous sequential IBIs for a sufficient duration to calculate HRV, although the estimates of HP over several seconds provided excellent convergence with the ECG signal.

### Hardware and Software

To obtain the criterion signals, a BIOPAC MP150 system (BIOPAC Systems, Inc., Camino Goleta, CA, USA) was used. The BIOPAC acquired the criterion data at a 1,000 Hz sampling frequency. The criterion signals acquired by the BIOPAC MP150 system were ECG from a 3-lead configuration on the chest, reflectance earlobe PPG (elPPG), and reflectance fingertip PPG (ftPPG). Breathing frequency was also assessed with a single strain gage respiration band.

To obtain the PhyC signal, a Grasshopper^®^ 03K2C IEEE-1394b (FireWire) digital camera (Point Grey Research Inc., Richmond, BC, Canada) was used. The digital camera serves as the sensor providing the signal from which the arterial pulse wave is extracted. Grasshopper^®^ digital camera monitors color signals with wavelengths between 350 and 750 nm, with a 640 × 480 pixel resolution and transmits the raw 8-bit RGB Bayer data at a sampling rate of approximately 60 frames per second. Subjects were positioned approximately 3 m in front of the camera.

Two software packages were used to acquire and to process the data. AcqKnowledge 4.2 (BIOPAC Systems, Inc., Camino Goleta, CA, USA) was used to collect and archive the BIOPAC data. This program output a text file containing five columns of information: relative time, analog physiological signals (i.e., elPPG, ECG, respiration), and the synchronization signal. LabVIEW™ System Design Software (National Instruments Corporation, Austin, TX, USA) was used to collect and to process the data from the color digital video camera. LabVIEW™ was also used to develop the applications to analyze, compare, and contrast the PhyC with the criterion signals.

### Data Quantification

Data quantification was performed at different levels of analysis, due to the hierarchical nature of parameter extraction. For instance, IBIs are first derived from the arterial pulse, and then components of HRV are derived from the IBI time series.

#### First Level: Raw Signal Processing, Synchronization, and Tasks Segmentation

##### PhyC—Pulse Extraction

For the offline analysis, images of a seated subject were captured by the camera at specified sampling frequency (approximately 60 Hz). The images were processed by an algorithm that selects a region of interest (ROI, section of the participant’s face) and separates the ROI into the RGB color planes. The mean values of each color component are calculated by a histogram function. Mean values of the blue color are not used because they contain the melanin information which varies with skin colors. As noted in Figure 1, mean values of green and red are less affected by differences in skin color across subjects. The mean values of the green and red color were divided (green/red) to create a common mode rejection ratio that minimizes common noise signals not related to arterial pulse (e.g., subject’s subtle movement, light shifts, and camera artifacts); the resulting signal was labeled Pulse_raw_ (37).

The 60 Hz raw values were paired with the time stamp of each frame to create a consecutive time series with inconsistent sampling frequency. This time series was then interpolated and up-sampled to create a constant 1 kHz signal (equivalent to the criterion signals). The inverse signal was calculated to resemble the volumetric changes of the pulse. More light absorption represents higher volume of blood in the arteries, and less light absorption lower volume of blood in the arteries. The signal was filtered using a second order Butterworth Band Pass filter (low cutoff = 0.5 Hz, high cutoff = 2.0 Hz). The cutoff frequencies were established to assure that different heart rate patterns between subjects could be detected. A healthy individual’s heart rate at rest is normally between 0.75 and 1.0 Hz (i.e., 60–80 beats per minute). The first derivative of the pulse signal was calculated to augment changes in the slope of the signal. These sequential steps enable the PhyC to generate an analog representation of the arterial pulse wave that is sampled at 1 kHz for synchronization with ECG and elPPG signals from the Biopac.

##### Ear Lobe Pulse (elPPG)

The elPPG was measured at a 1 kHz through a DC amplifier (no filter settings) to preserve the slower aspects of signal reflecting the sympathetic influences on vasomotor tone. To extract the pulse peak, the elPPG signal was centered on zero by subtracting the first value to the entire trend, next it was filtered using a second order Butterworth Band Pass filter (low cutoff = 0.5 Hz, high cutoff = 2.0 Hz). A first derivative function was applied to translate the signal into slope changes over time and stabilize the estimation of pulse arrival times.

##### Electrocardiogram

The ECG was sampled at a 1 kHz using the preferred ECG BIOPAC MP-150 analog band-pass filter settings with a gain of 1,000.

##### Data Segmentation by Task

A time log file documented the start and end of each challenge for each participant and enabled synchronization with the physiological variables. ECG, elPPG, and the resampled (1 kHz) PhyC were aligned by use of a synchronization pulse in the Biopac data. The time log was used to extract the physiological signals associated with the different conditions of the experiment. In this article, segments corresponding to the following tasks were analyzed: initial baseline (1BSL), SDB, rest (2BSL), shallow rapid breathing (RB), and final baseline (4BSL) for ECG, elPPG, and PhyC. Due to variations in tolerance, movement, and the time course of responding, the CP task was not analyzed.

#### Second Level: Extraction of the IBIs

##### R Peak and Peak of Pulse Wave Detection

To accurately extract the heart rate pattern, a cardio peak-valley detector (CPVD) was developed. The CPVD is a LabVIEW™ based algorithm that extracts peaks or valleys of different physiological waves, such as the ECG, PPG, and respiration. The CPVD is able to detect peaks or valleys (time position and amplitude) of the signal by an adaptive approach. The algorithm uses a window of data to find a peak, and once the peak is detected the window moves one step and looks into the next window for the next peak. Each window is set to contain at least one physiological peak. For example, the window width for the ECG is around 700 ms and the step size is around 400 ms to assure that and R peak would be present in the analyzed window. For the first three peaks, the window width and step sizes are constant values, after the third peak is extracted, the information of the last two peaks time difference is used to adapt the window width and step size. This ensures that the window width and step size will adapt to the individual’s response to the task challenge. If the heart rate increases, the window and step sizes decrease. If the heart rate decreases, the window and step sizes increase. Within each window, a peak detection algorithm is applied.

The peak detection algorithm applied a quadratic fit to identify peaks above a specified threshold determined by the distribution of samples within the window. The peak detector algorithm fits a parabola to a sequence of successive points assuming a specified width pulse wave or the R-wave of the ECG signal. The algorithm checks whether each parabola is at the local maxima by evaluating the sign of the quadratic coefficient, which indicates the parabola’s concavity. The number of data points used in the ECG fit is specified by a width of approximately 15 ms. Each peak resulting within the window is tested against the threshold. Peaks with heights lower than the threshold are ignored. Because the algorithm calculates all the peaks above the threshold, it is possible to find two or more peaks within a window, in that case the first peak is compared against the maximum within the window and the one with the greater amplitude is selected as the peak of that window. Because the peak detection algorithm uses a quadratic fit to find the peaks, it functionally interpolates between the data points. Therefore, the timing precision of the peak location exceeds the precision of the original sampling rate of the signal.

The CPVD generates a trend formed by pairs with coordinates of time and peak amplitude. The CPVD has been tested in the analysis of several independent physiological signals, resulting in less than 2% missing peaks for signals with few artifacts. The CPVD has also performed well in extracting peaks from data with periods of low signal to noise ratio (37). The CPVD was used to calculate the R-peaks of the ECG and the pulse wave peaks of the elPPG and the PhyC during the different tasks.

##### Interbeat Interval

Interbeat interval is the time between consecutives heart beats, expressed in milliseconds. IBIs are calculated by the consecutive differences of the time component of the R-peaks or the pulse peak coordinates.

#### Third Level: Quantification of HRV Parameters

##### Time Sampled Mean IBIs from 2 and 5 s Windows (IBI 2sW and IBI 5sW)

The IBI event series was resampled at 2 Hz to generate an equally spaced intervals time series. The 2 Hz time sampled estimates of HP were used for calculating HP, RSA, and LF during each experimental condition and for calculating HP estimates for sequential 2- and 5-s windows. The HP is the average value of the 2 Hz IBI time series within a specific segment or task.

##### Respiratory Sinus Arrhythmia

Based on the Porges–Bohrer method (38, 39) a third-order, 21-point moving polynomial filter (MPF) was applied to the 2 Hz IBI time series to remove low frequency oscillations and slow trend. The residual detrended output of the MPF was filtered with a Kaiser FIR windowed filter with cutoff frequencies that remove variance not related to spontaneous breathing in adults (0.12–0.40 Hz). The filtered detrended output was divided into sequential 30-s epochs and the variance within each epoch is transformed by a natural logarithm [ln(ms^2^)], the mean of these epoch values is used as the estimate of RSA for the specific segment.

##### LF

Based on the Porges–Bohrer method (38, 39) a third-order, 51-point MPF was applied to the 2 Hz IBI trend to remove extremely low frequency oscillations and slow trend. The residual detrended output of the MPF was filtered with a Kaiser FIR windowed filter with cutoff frequencies (0.04–0.10 Hz). The filtered detrended output was divided in 30 s epochs and the variance within each epoch is transformed with a natural logarithm [ln(ms^2^)], the mean of the epochs values is used as an estimate of LF for the segment.

### Data Analysis

Statistical analyses were performed using IBM SPSS Statistics for Windows, Version 24.0. Armonk, NY, USA: IBM Corp.

#### Bland–Altman (B–A) Plots

Bland–Altman plots were used to compare the PhyC generated IBI values with the IBI values generated by the criterion signals (ECG and elPPG) and also to compare values generated by the elPPG and ECG signals. B–A plots enable the determination of agreement between two sensors, by plotting the mean between pair of measurements against its difference. Visual inspection of the B–A plots was used to identify systematic biases and possible outliers. Paired *t*-tests evaluated whether the differences between the signals were biased (i.e., one signal source generating longer or shorter values). B–A plots and the *t*-test were performed on IBIs collected from all participants during all tasks.

#### Scatter Plots and Linear Regressions

Scatter plots and linear regression analyses were used to visualize and calculate the level of convergence between the physiological measures derived from PhyC with each of the criterion signals (ECG and elPPG) and between the two criterion signals. Parameters from Mukaka’s (40) paper where used to interpret the size of the correlation coefficients.

#### Size of Effect Repeated Measures ANOVA (RMANOVA)

Value of the partial eta-squared for the TIME effect, obtained by RMANOVA (General Linear Model), of the HP, RSA, and LF for each sensor across the five tasks was used to evaluate if the effect size of the experimental manipulations observed by the three sensors were in the same magnitude and of the same level of significance.

## Results

### The PhyC Signal

**Figure 2:**
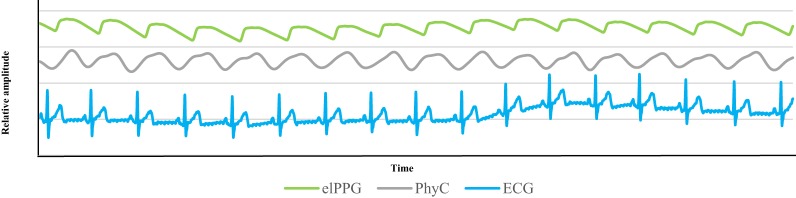
Physiological signals. Plot of 15 s of data for participant #01 during 1BSL, the green line represents the elPPG, the gray line is the PhysioCam (PhyC), and the blue line is the electrocardiogram (ECG).

The PhyC produces a physiological signal that resembles the one obtained by the elPPG as observed on the synchronized 15 s segment of data shown in Figure 2, elPPG is the top green line and the PhyC is the middle gray line, and the ECG the bottom blue line. The PhyC signal does not look as stable as the traditional elPPG, but follows its pattern. The PhyC signal shows the same offset from the ECG as the elPPG.

### B–A Plots

**Figure 3:**
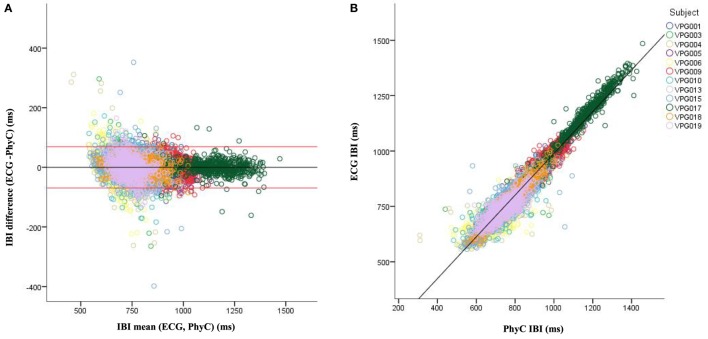
Bland–Altman and scatter plot for interbeat interval (IBI) from the electrocardiogram (ECG) and PhysioCam (PhyC), color coded by participant. **(A)** Plot of the IBI differences vs the means for the ECG and PhyC. Red lines indicate the 95% confidence interval. **(B)** Scatter plot of the PhyC vs ECG IBIs.

**Figure 4:**
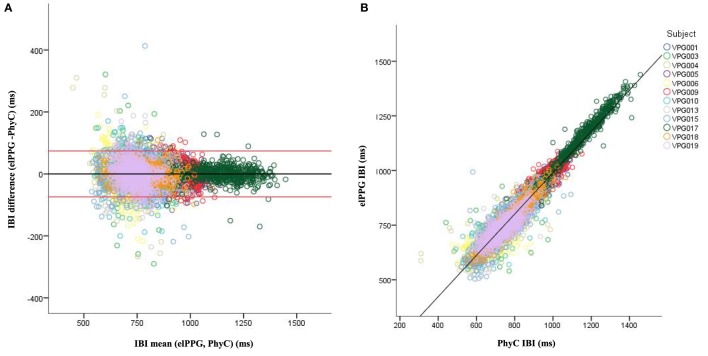
Bland–Altman and scatter plot for interbeat interval (IBI) from the elPPG and PhysioCam (PhyC), color coded by participant. **(A)** Plot of the IBI differences vs the means for the elPPG and PhyC. Red lines indicate the 95% confidence interval. **(B)** Scatter plot of the PhyC vs elPPG IBIs.

**Figure 5:**
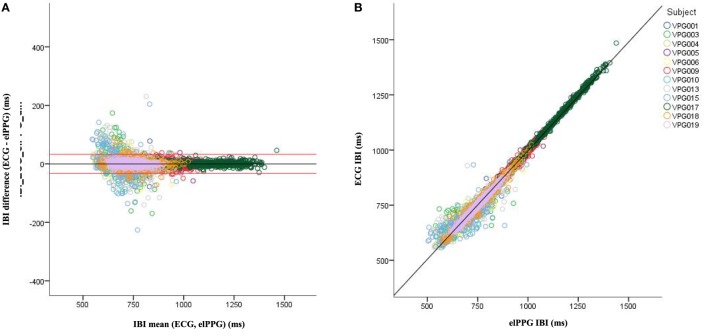
Bland–Altman and scatter plot for interbeat interval (IBI) from the electrocardiogram (ECG) and elPPG, color coded by participant. **(A)** Plot of the IBI differences vs the means for the ECG and elPPG. Red lines indicate the 95% confidence interval. **(B)** Scatter plot of the elPPG vs ECG IBIs.

Visual inspection of the B–A plots located in the A panels of Figures 3–5 indicate excellent agreement and minimal bias between the sequential IBIs measured with ECG and PhyC (color coded by participant) in Figure 3, elPPG and PhyC in Figure 4, and ECG and elPPG in Figure 5. For the three cases, the B–A plots suggest that error magnitude is slightly larger for shorter IBIs, the IBI differences are larger on the left side of the B–A plots and closer to zero on the right side. The 95% confidence intervals are listed in Table 1. Note that the mean of the differences are less than 0.1 ms and that there are no significant differences between the metrics in central tendency. The *t*-test results confirm that the pairs of sensors are measuring the same parameter, mean of the differences are not significantly different than zero, indicating that there is no fixed sensor bias.

**Table 1:** Bland–Altman (B–A) contrast parameters for interbeat interval between sensors.

B–A contrast	95% CI (ms)	Mean of the differences (ms) (SD)	Mean of the differences single sample *t*-test
Electrocardiogram (ECG) vs PhysioCam (PhyC)	±69	−0.07 (35.3)	*t* (8,574) = −0.17, *p* = 0.86
elPPG vs PhyC	±74	−0.02 (37.8)	*t* (8,574) = −0.04, *p* = 0.97
ECG vs elPPg	±33	−0.08 (16.7)	*t* (8,574) = −0.45, *p* = 0.65

*Confidence interval, mean of the differences between sensors, single sample t-test of the mean differences*.

Scatterplots with regression analyses contrasting the sensor pairs are illustrated in the B panels of Figures 3–5. The regression models provide excellent fits to the IBI data with *R*^2^ above 0.90 as shown in Table 2.

**Table 2:** Regression model parameters between sensors when measuring interbeat interval.

Sensors	Regression model	*R*^2^
Electrocardiogram (ECG) vs PhysioCam (PhyC)	*Y* = 1.00*X* + 47.65	0.94
elPPG vs PhyC	*Y* = 0.95*X* + 44.84	0.93
ECG vs elPPg	*Y* = 0.99*X* + 12.5	0.99

**Figure 6:**
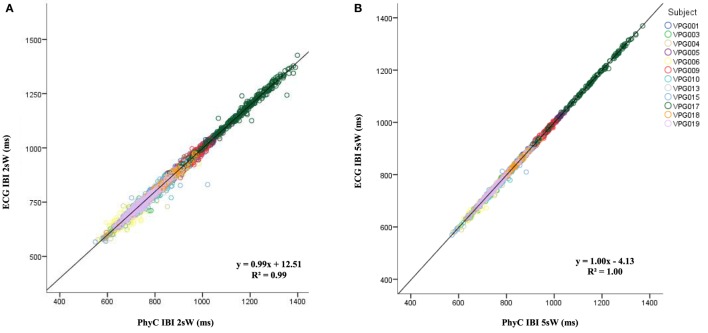
Scatter plot for average window interbeat interval (IBI) from the electrocardiogram (ECG) and PhysioCam (PhyC) color marked by participant. **(A)** Scatter plot of the PhyC vs ECG for 2 s average windows IBIs. **(B)** Scatter plot of the PhyC vs ECG for 5 s average windows.

To stabilize the estimates from the PhyC, IBI data were averaged within 2 and 5 s windows. Figure 6 illustrates the scatterplots and regression fits for the time windowed estimates of ECG- and PhyC-derived IBI data. Note relatively perfection convergence (i.e., *R*^2^ = 1.0) and reduction in dispersion (i.e., reflected in the SD of the differences) as the window for estimating IBI increases from beat-to-beat to 2 s, and then to 5 s.

As observed in Table 3, as the window to average IBI measures widens from individual IBIs to 2 and 5 s windows, the SD of the differences between the PhyC and the ECG decreases and the *R*^2^ increases reaching unity. The IBI data by subject by sensor shown in Table 4 are consistent with the scatter plots of Figure 6, in which averaging the IBI within a 2 and 5 s window considerably improves the linear regression between the PhyC and both the ECG and the elPPG for all subjects.

**Table 3:** Differences mean and SDs for all subjects, all consditions between the electrocardiogram (ECG) and PhysioCam (PhyC) for interbeat interval (IBI), IBI 2 s windows, IBI 5 s windows, and HP 30; and *R*^2^ of linear regression for PhyC on ECG.

		Differences		Linear regression
PhyC vs ECG	*N*	Mean	SD	*R*^2^
IBI	8,574	−0.07	35.26	0.94
2 s window IBI	3,501	−1.51	13.03	1.00
5 s window IBI	1,403	−1.50	5.67	1.00
Heart period (30 s epoch)	60	−1.69	1.61	1.00

**Table 4:** *R*^2^ of the interbeat interval (IBI) linear regression between sensors by subject considering all the IBI, 2sW, and 5sW.

Subject	Sensors	NO window	2 s window	5 s window
VPG001	ECG_PhyC	0.84	0.96	0.99
	elPPG_PhyC	0.83	0.96	0.99
	ECG_elPPG	0.99	1.00	1.00

VPG003	ECG_PhyC	0.73	0.96	0.99
	elPPG_PhyC	0.64	0.94	0.99
	ECG_elPPG	0.86	0.97	0.99

VPG004	ECG_PhyC	0.69	0.94	0.98
	elPPG_PhyC	0.67	0.94	0.98
	ECG_elPPG	0.96	0.99	1.00

VPG005	ECG_PhyC	0.91	0.98	1.00
	elPPG_PhyC	0.91	0.98	1.00
	ECG_elPPG	0.99	0.99	1.00

VPG006	ECG_PhyC	0.64	0.93	0.98
	elPPG_PhyC	0.65	0.94	0.99
	ECG_elPPG	0.94	0.98	0.99

VPG009	ECG_PhyC	0.80	0.94	0.99
	elPPG_PhyC	0.80	0.94	0.98
	ECG_elPPG	0.98	0.99	1.00

VPG010	ECG_PhyC	0.69	0.94	0.99
	elPPG_PhyC	0.66	0.94	0.99
	ECG_elPPG	0.93	0.99	1.00

VPG013	ECG_PhyC	0.88	0.98	1.00
	elPPG_PhyC	0.79	0.94	0.98
	ECG_elPPG	0.89	0.96	0.98

VPG015	ECG_PhyC	0.81	0.96	0.99
	elPPG_PhyC	0.78	0.96	0.99
	ECG_elPPG	0.92	0.99	1.00

VPG017	ECG_PhyC	0.96	0.99	1.00
	elPPG_PhyC	0.96	0.99	1.00
	ECG_elPPG	1.00	1.00	1.00

VPG018	ECG_PhyC	0.90	0.98	1.00
	elPPG_PhyC	0.87	0.98	0.99
	ECG_elPPG	0.97	0.99	1.00

VPG019	ECG_PhyC	0.65	0.95	0.99
	elPPG_PhyC	0.64	0.95	0.99
	ECG_elPPG	0.98	0.99	1.00

*p Values < 0.05*.

Table 4 documents a range of individual differences in which PhyC estimates individual IBIs better in some participants, *R*^2^ values ranging from 0.64 to 0.96 when compared to ECG. However, these differences are minimized with a 2 s window (i.e., *R*^2^ values range from 0.93 to 1.0) and virtually disappear with a 5 s window (i.e., *R*^2^ values range from 0.98 to 1.0).

**Figure 7:**
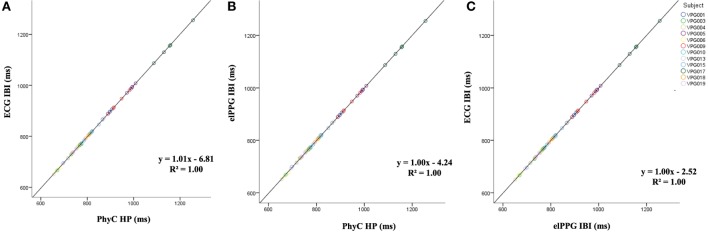
Scatter plots between sensors for heart period (HP), color coded by participant. **(A)** PhysioCam (PhyC) vs electrocardiogram (ECG). **(B)** PhyC vs elPPG. **(C)** elPPG vs ECG.

Figure 7 indicates convergence around the expected model *y* = *x* for all the sensor comparisons for the measures of HP with *R*^2^ of 1.00 for the three cases.

**Figure 8:**
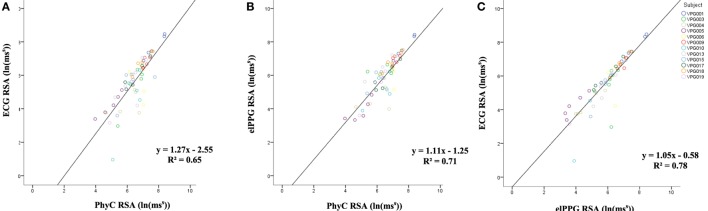
Scatter plots between sensors for respiratory sinus arrhythmia (RSA), color coded by participant. **(A)** PhysioCam (PhyC) vs electrocardiogram (ECG). **(B)** PhyC vs elPPG. **(C)** elPPG vs ECG.

The linear regression of RSA between ECG and PhyC from Figure 8A while not in convergence with the model *y* = *x* (*y* = 1.27*x* − 2.55) indicates a moderately strong correlation *R*^2^ of 0.65. The linear regression of RSA between elPPG and PhyC from Figure 8B while not in convergence with the model *y* = *x* (*y* = 1.11*x* − 1.25) indicates a moderately strong correlation *R*^2^ of 0.71. The strongest correlation is observed between ECG and elPPG as shown in Figure 8C with *R*^2^ of 0.78, close to the 0.8 threshold to be considered a strong correlation.

**Figure 9:**
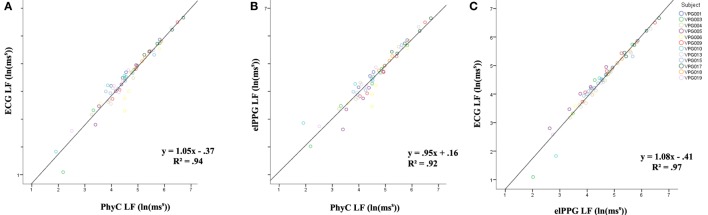
Scatter plots between sensors for LF, color coded by participant. **(A)** PhysioCam (PhyC) vs electrocardiogram (ECG). **(B)** PhyC vs elPPG. **(C)** elPPG vs ECG.

Figure 9 depicts the linear regression on LF between the different sensors (Figure 9A) ECG and PhyC, (Figure 9B) elPPG and PhyC, and (Figure 9C) ECG and elPPG. The three models show convergence around the expected model *y* = *x*, with the elPPG and ECG showing the strongest *R*^2^ of 0.97 and the PhyC and elPPG showing the weakest with *R*^2^ of 0.92.

The RMANOVA is used to demonstrate the sensitivity to change across time in each HRV parameter, as observed by each sensor. Results of the partial eta-squared for time in the RMANOVA are shown in Table 5. The three sensors measure similar, significant time effects for HP. All three sensors measure a similar, but not significant time effect for LF. While measuring RSA, the PhyC captures a significant, but reduced, effect for time as compared to the ECG, while the elPPG fails to detect a significant time effect for the parameter.

**Table 5:** Effect size within sensor by heart rate variability parameter.

Effect size	Electrocardiogram	elPPG	PhysioCam
Heart period	0.23*	0.22*	0.22*
Respiratory sinus arrhythmia	0.39*	0.14	0.28*
LF	0.15	0.16	0.18

*p Values < 0.05.

### Change Scores for HP, RSA, and LF

**Figure 10:**
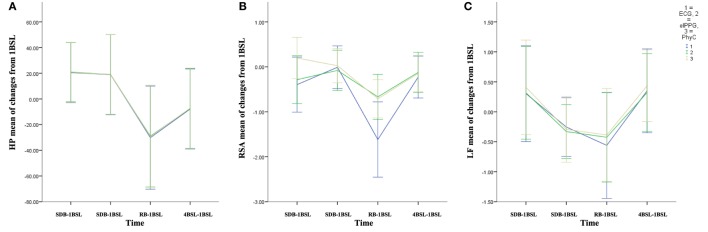
Mean of changes from the 1BSL (error bars: ±2 SE) for the three sensors electrocardiogram (ECG), elPPG, and PhysioCam (PhyC). **(A)** Heart period (HP). **(B)** Respiratory sinus arrhythmia (RSA). **(C)** LF (error bars: ±2 SE).

Change scores shown in Figure 10 indicate that the PhyC and the elPPG are able to track the change scores from 1BSL obtained with the ECG (criterion signal sensor) for the HP parameter. While the PhyC and the elPPG do not track the change scores for RSA obtained with the ECG, they both reflect a similar, attenuated response for RSA across time. Both sensors track the moderate changes of LF observed by the ECG.

## Discussion

The PhyC reliably tracks IBIs when compared to either criterion signal (i.e., ECG or elPPG). As the window of comparison expands from individual heartbeats to 2 and 5 s sequential windows, the modest differences between sensors decreases dramatically and both the PhyC and elPPG converge with the ECG-derived values. Thus, the peripheral measures capture the slower dynamics in heart rate almost exactly, while making small errors in the rapid changes in IBI from beat to beat.

Inspecting the B–A plots reveals greater error in estimating IBIs with either the PhyC or elPPG when IBIs are shortest. Some of this error can be attributed to the shift in signal to noise ratio as the constant measurement error occupies a greater percentage of the interval defining the IBI.

The physical constraints of vascular transmission of the pulse wave also contribute to the deviations between the ECG and the peripheral measures of IBI (elPPG and PhyC). The cardiovascular system acts as a filter, modulating the duration between pulses that define IBIs and the parameters of this filter are non-constant due the neural regulation of vascular tone which has known oscillatory components (41). The conclusion that vascular transmission impacts short-term variation in IBIs is strengthened by: (1) the observation that similar dispersion in deviations from ECG-derived IBIs were observed when comparing elPPG with the ECG (signals collected with the same device at the same sampling frequency) and (2) the fact that similar outcomes have been reported for PPG (42). The error manifested differently across comparisons with the ECG-derived HRV metrics quantified in this study: (1) HP was virtually devoid of error with linear regression documenting the convergence among the HP estimates derived with PhyC, elPPG, and ECG during 30-, 5-, and 2-s epochs; (2) RSA calculation showed modest error magnitude with larger errors during segments with shorter IBIs, as illustrated during the RB condition when RSA estimates from both PhyC and elPPG show similar deviation from the ECG measure; (3) LF was virtually devoid of error documenting that slower components of HRV are relatively immune to the dampening effects of the vascular system.

Other sources of error are due to the physics of the sensor embedded in the PhyC system. Compared to the contact PPG, the PhyC is more sensitive to movements and lighting shifts in the environment, although both measures are based on light absorbance at similar wavelengths. Since the PhyC and the elPPG measure cardiovascular activity at the periphery it would be expected to find more agreement between them then when either is compared individually to the ECG. However, this was not the case with regression analyses of the IBIs suggesting the presence of an additional source of error, likely due to the frame rate consistency of the camera or the complexities of the algorithm used in the PhyC system. The regression analyses between IBIs was slightly stronger between elPPG and ECG (*R*^2^ 0.99) than when comparing PhyC-derived IBI values with either the elPPG or ECG (*R*^2^ 0.94). In future embodiments, the discrepancy between PhyC and elPPG may be minimized by improving the stability of the sensor system (e.g., optimized Bayer mapping wavelength selection, improved motion tracking) and increasing the sampling rate.

Lessons learned from this study provide guidance for planned improvements to PhyC: (1) reduce movement error by improving the face tracker either through an embedded processor in the camera, external software analysis of the image frames, or a parallel tracking system based on additional sensors, (2) fine tune the selection of the pixels within each frame that contain the physiological information of interest, (3) maximize sensitivity across the range of skin colors by expanding the camera color spectrum to different wavelengths, and (4) use more powerful processors to facilitate the extraction of the pulse signal, enabling online extraction in real time. A combination of these hardware and software modifications will improve the PhyC performance as development continues. Nevertheless, optics of the sensor and available light in the environment (i.e., photons) are key elements in identifying and limiting applications of the PhyC.

In our study, we documented that the PhyC tracks IBI, HP, and LF with sufficient accuracy and precision to be used instead of the traditional contact devices when measuring those components of HRV. However, when measuring HRV from the periphery, the peripheral vascular activity influences the pulse-to-pulse intervals. Although the central tendency (i.e., mean) of pulse-to-pulse intervals converge with the R–R intervals (i.e., IBIs) generated from the ECG, on a beat-to-beat level, vascular rhythms and responses result in variations in the coherence between the two signals. Since the vascular rhythms are frequently slower than RSA, these rhythms tend to blunt the dynamic changes in RSA. This poses the question of whether the pulse-to-pulse intervals can be corrected to account for the vascular filtering effects? The frequency-dependent nature of components of the error suggests that a dynamic algorithm could be developed to adapt to the modulation effect of the cardiovascular system and reduce the rhythmic sources of error. In its current embodiment, PhyC can generate HRV measures from the periphery pulse that can be used interchangeably with those obtained from the ECG. The current PhyC system is capable of HRV estimation when the subject is seated and breathing normally, circumstances that cover a wide range of clinical and research demands. As long as the face is visible to the photosensor, future iterations of the PhyC will be able to deal with greater body movements.

The PhyC provides several advantages over contact measurement of heart rate: (1) the ability to measure HRV by non-contact sensors permits observation of a more neutral baseline by eliminating stressful disruptions caused by placing contact sensors on the participant, (2) the planned ability to measure several participants with the same sensor following further developments on the face tracking algorithm, (3) the collection of additional vascular signals, including sympathetic regulation of vasomotor tone, which are not available in ECG-derived measures, and (4) the system ability to work online in near real time to provide instantaneous measurement and continuous feedback.

These findings provide the basis needed to explore applications of this new methodology in psychophysiological and biomedical research as well as in applied settings. Expanding our understanding of the science behind the PhyC, which includes neurophysiological regulation of the cardiovascular system, sensor design, feature extraction, and algorithm development, suggests that an optimized system can extract, quantify, and interpret the neural regulation of the heart and the peripheral vascular system from the optical information collected passively from a subject. The system will continue to expand to accurately recreate the sensitivity and specificity of the ECG and eventually to quantify additional physiological parameters of interest to researchers, doctors, and commercial enterprises interested in neural regulation of the cardiovascular system.

## Ethics Statement

This study was carried out in accordance with the recommendations of the Institutional Review Board of the University of Illinois at Chicago with written informed consent from all subjects. All subjects gave written informed consent in accordance with the Declaration of Helsinki. The protocol was approved by the Institutional Review Board of the University of Illinois at Chicago as protocol # 2012-0206 entitled “Real Time Non-contact Extraction of Human Arterial Pulse.”

## Author Contributions

The manuscript is based on MD PhD dissertation work, under SP mentoring, and GL technical guidance. MD, GL, and SP conceived and designed the PhysioCam. MD and GL performed the data acquisition, processing, and statistical analyses. MD, GL, and SP contributed to the selection of the different metrics evaluated in the manuscript and interpreted the results. The intellectual content of the manuscript was jointly created and approved by MD, GL, and SP. All three authors drafted the text, with MD providing the original framework, and all three agreed to be accountable for all aspects of the work.

## Conflict of Interest Statement

The authors declare that the research was conducted in the absence of any commercial or financial relationships that could be construed as a potential conflict of interest.
